# Non-tuberculous mycobacterial disease in a high TB/HIV burden setting

**DOI:** 10.5588/ijtldopen.25.0205

**Published:** 2025-07-09

**Authors:** S. Janssen, Z. Ismail, T. Lovelock, S. Hugo, M. van Schalkwyk, A. Parker, B.W. Allwood, C.F.N. Koegelenberg, J. van Ingen, A. van Laarhoven, T. Mabuka, H. Prozesky, J. Taljaard

**Affiliations:** ^1^ TASK, Cape Town, South Africa;; ^2^ Radboud University Medical Center, Nijmegen, The Netherlands;; ^3^ Department of Medicine, Stellenbosch University and Tygerberg Hospital, Cape Town, South Africa.

**Keywords:** tuberculosis, NTM, South Africa, *M*. *avium*, *M. kansasii*, *M. abscessus*

Dear Editor,

Non-tuberculous mycobacterial (NTM) disease is an expanding global problem, posing challenges for healthcare systems due its complex diagnosis and treatment.^1^ NTM pulmonary disease (NTM-PD) is mostly associated with structural lung disease like COPD, bronchiectasis, or post pulmonary TB (PTB). *Mycobacterium avium* complex (MAC) bacteria are the leading cause of NTM-PD, although infections by rapidly growing *M. abscessus* are increasing.^1^ Clinical practice guidelines exist for diagnosing and treating NTM-PD, requiring clinical, radiologic, and microbiologic criteria, with exclusion of other diagnoses.^2^ Treatment entails species-specific combinations of antibiotics, for an average of 18 months.^2^ In people living with HIV (PLHIV) disseminated NTM disease is associated with advanced immunodeficiency and optimizing antiretroviral therapy is crucial. Data on NTM disease in sub-Saharan Africa remains scarce, with distinct epidemiology linked to post-TB lung disease (PTLD) and HIV.^3,4^ Laboratory-based studies report frequent positive sputum cultures with NTM among TB-suspected patients, though it is unclear if these indicate colonization or disease.^5,6^

This retrospective cohort study aims to explore the epidemiology and outcomes of NTM disease patients at a tertiary care center in Cape Town, South Africa. Patients referred to the Adult Infectious Diseases or Pulmonology Clinics at Tygerberg Hospital with confirmed or suspected NTM disease between January 2013 to December 2022 were included. Tygerberg Hospital serves a population of 3.4 million, in a region with high HIV and TB burdens. Patients were identified through standard of care clinic databases. Clinical information was extracted from patient files, national and provincial databases and captured using a REDCap database.^7^ Chest radiograph data was collected with a standardized tool scored by a physician for presence of cavities, infiltrates, adenopathy, effusions, and lung field involvement. International case criteria for NTM disease were followed.^2^ Treatment outcomes followed NTM Network European Trialsgroup (NET) definitions, with main outcomes being survival status at study end or discharge, and culture status six months post-treatment initiation.^8^ Descriptive statistical analysis used R Studio and Excel, with χ^2^ tests for categorical data, Student’s t-test for parametric continuous data, and Mann-Whitney-U tests for non-parametric data.

Stellenbosch University’s Health Research Ethics Committee approved the study (reference N23/05/050), informed consent was waived due to its retrospective nature.

We identified 59 patients with NTM disease, mostly male with a median age of 42 years (Table). Two-thirds were PLHIV with advanced immunodeficiency and 71% (27/38) were not virologically suppressed. A history of TB was common, though only a minority had microbiologic confirmation. Smoking was a prominent risk factor. Fifty patients (85%) had NTM-PD, with 74% (37/50) showing cavitary disease and 24% (12/50) a previous diagnosis of NTM-PD. Of 9 patients with extrapulmonary disease, 6 had disseminated disease. *M. avium* complex was the most frequent causative species, followed by *M. kansasii* and *M. abscessus*. Sputum microscopy was positive in 56% (37) of pulmonary cases. Drug susceptibility testing (DST) was only performed in case of treatment failure, with macrolide resistance found in 5 patients (4 with *M. intracellulare* and 1 with *M. abscessus* pulmonary disease) and aminoglycoside resistance in 1 (*M. intracellulare*) (see Table).

**Table.** Baseline characteristics, diagnosis and treatment by culture status at 6 months.

Demographics	All patients (n=59)	Culture positive at 6 months (n=22)	Culture negative at 6 months (n=19)	p-value
Male sex (n,%)	40 (68)	17 (77)	15 (79)	1.00
Age (years; median, IQR)	42 (33–52)	38.5 (32.3–42.4)	42 (37.5–58.5)	0.30
BMI (kg/m^2^; median, IQR)	18.8 (7.25–24.0)	17.5 (10.8–27.0)	18.9 (16.8–23.3)	0.21
*Risk factors*				
Living with HIV (n,%)	38 (64)	13 (59)	9 (47)	0.27
CD4 (cells/microliter; median, IQR)	40 (13–158)	34 (17–383)	24 (12–86)	0.39
HIV viral load (copies/mL)	6024 (0–78487)	11,384 (1506–430847)	202 (0–43722)	0.53
HIV viral load detectable (n/N HIV positive)	27/38 (71)	10/13 (77)	6/9 (67)	0.69
*ART status at time of NTM diagnosis (n,%)*				
ART naïve	2 (5.2)	1 (8)	1 (11)	0.28
On ART	33 (87)	12 (92)	6 (67)	
ART interrupted	3 (7.9)	0 (0)	2 (22)	
TB diagnosis in history	45 (76)	20 (91)	14 (74)	0.22
Microbiologically proven TB	19 (32)	8 (36)	6 (32)	0.92
*Other*				
Smoking	29 (49)	12 (55)	12 (63)	0.25
Ethanol abuse*	4 (7)	2 (9)	2 (11)	0.93
COPD	3 (6)	1 (5)	1 (5)	1.00
Bronchiectasis	10 (17)	6 (27)	2 (11)	0.25
Diabetes	2 (3.4)	1 (5)	0 (0)	1.00
GERD	3 (6)	1 (5)	1 (5)	1.00
Use of systemic immunosuppressive drugs	1 (1.7)	1 (5)	0 (0)	1.00
*Diagnosis*				
Duration of symptoms (months, median, IQR)	12 (7.25–24)	19 (10.8–27.0)	7 (5.0–9.7)	0.31
Median time to treatment initiation (days; median, IQR)	198 (57–804)	610 (130–1713)	128 (61–256)	0.01
Total number of positive cultures (median, IQR)	11.5 (7–18)	17 (12–26)	13 (9–18)	0.04
Number of positive cultures before diagnosis (median, IQR)	3	6 (3–10)	2.5 (2–3)	<0.01
DST performed at baseline (n, %)	0	0 (0)	0 (0)	NA
Genotypic DST during treatment (n, %)	15 (25)	10 (45)	2 (11)	0.02
Macrolide resistance detected (n, %)	6 (40)	4 (40)	1 (50)	1.00
Phenotypic DST during treatment (n, %)	6 (10)	4 (18)	1 (5)	0.35
*Chest X–rays*				
Cavities of > 4cm in aggregate at baseline (n,%)	14 (24)	7 (32)	5 (26)	0.68
Disease extent of more than one hemihorax at baseline (n,%)	8 (14)	3 (14)	2 (11)	0.18
Disease extent of more than one hemihorax at study end (n,%)	17 (28)	10 (45)	3 (16)	0.15
*Treatment*				
Treatment Duration (days; median, IQR)	470 (143–720)	602 (401–1002)	523 (484–772)	0.22
<3 agents used at treatment initiation (n,%)	19 (32)	10 (45)	3 (16)	0.05
Second line aminoglycosides (n, %)	11 (19)	9 (41)	1 (5)	0.01
*Outcomes*				
Deceased (n, %)	20 (34)	11 (50)	5 (26)	0.11
Treatment failure (n, %)	4 (7)	3 (14)	1 (5)	
Unknown outcome (n, %)	22 (37)	6 (27)	5 (26)	
On treatment (n, %)	4 (7)	0 (0)	3 (16)	
Cured (n, %)	9 (15)	2 (9)	5 (26)	

Baseline characteristics, diagnosis and treatment outcomes are shown for all patients combined, and for the subset with cultures available at 6 months, those whose sputum cultures remained positive at 6 months and those whose sputum cultures converted to negative 6 months after treatment (minimum of 2 consecutive negative cultures). P-values are shown for the comparison between 6-month culture status groups. *Ethanol abuse defined as > 28 units/week for males; > 21 units/week females. IQR = interquartile range; BMI = body mass index; VL = viral load; ART = antiretroviral therapy; COPD = chronic obstructive pulmonary disease; GERD = gastro-esophageal reflux disease; DST = drug susceptibility testing; LTFU = lost to follow up; IDC = infectious diseases clinic.

Patients had a median symptom duration of 12 months, and the median time from first positive culture to treatment initiation was 198 days. Common symptoms included cough (38/59, 64%), sputum production (22/59, 37%), and weight loss (26/59, 44%). Most patients began treatment targeted at the causative NTM species with three or more drugs, typically azithromycin, ethambutol, and rifampicin (Table). Second-line treatments included aminoglycosides, quinolones, and clofazimine. One patient underwent a pneumectomy because of treatment failure. Culture conversion at six months occurred in 46% of patients, most commonly with *M. kansasii* (86%). Patients with persistent positive cultures had longer durations to treatment initiation and initiated regimens with less than 3 drugs more frequently (Table). Mortality was 34% (20/59), with a median age of 43 years (interquartile range (IQR) 38-56) and a median follow-up of 33 months (IQR 13–48). Patients who died had longer intervals between first positive culture and treatment initiation (median 269 days (IQR 49–1104) vs. 121 days (IQR 58–707), p<0.001), tended to have a longer duration of symptoms at presentation (median 24 months (IQR 16.5–30) vs. 12 months (IQR 5–14), p=0.85) and initiated treatment with less than 3 drugs more frequently (9/20 (45%) of those who died vs 10/39 (26%) of those who survived, p=0.15). Persistent positive cultures despite 12 months of treatment occurred in 8% of survivors, with one achieving cure post-pneumectomy. Loss to follow-up (LTFU) affected 37% (22/59) of patients, and by study end, 4 (7%) were still on treatment and 9 (15%) were cured. Radiologic progression was noted, with increased disease extent in 34% of patients at final follow-up.

This retrospective study highlights poor treatment outcomes for patients with NTM disease, including high rates of treatment failure, mortality and LTFU. HIV infection and smoking were common risk factors, and many patients had a history of presumed PTB, although less than half had microbiologic confirmation, suggesting possible misdiagnosis of NTM-PD as PTB. Our findings differ from studies in high-income settings, where structural lung diseases like COPD and malignancies are common risk factors for NTM-PD.^9–11^ In this study, most patients were PLHIV with advanced immunodeficiency. Unlike prior reports, where isolated pulmonary disease was rare in PLHIV, the majority of our cohort had NTM-PD without signs of dissemination.^12^ Mortality rates were comparable to a Danish study, but patients in our study died at a much younger age (median 43 vs. 72 years).^13^ Previous PTB is a risk factor for NTM disease in patients from TB endemic regions.^11,14^ However, this may be overestimated in cases where diagnosis is based on a clinical diagnosis with positive AFB microscopy only. The high prevalence of presumed PTB without microbiologic confirmation and long symptom duration before NTM diagnosis suggests frequent misdiagnosis and diagnostic delays. This has also been reported in other settings with high TB incidence.^6^ The shift from sputum microscopy and culture to newer diagnostic algorithms relying on Xpert and urine LAM^15^ further reduces opportunities to identify NTM disease. NTM disease should be considered in patients with suspected TB relapse or non-resolving TB. The species distribution in this study aligns with global data, with *M. avium* complex as most common causative agent.^1^ Culture conversion was less likely in patients treated with fewer than three drugs, and 42% of those did not meet criteria for this approach due to cavitary disease.^2^

This study is limited by its retrospective design, small sample size, and lack of clinical outcome measures and causes of mortality. High LTFU rates illustrate challenges of managing NTM disease in resource-limited settings, where diagnostic delays contribute to severe lung damage and limited clinical improvement.

In conclusion, NTM disease in this South African cohort was associated with high mortality and LTFU. Improved diagnostic strategies and increased awareness among healthcare providers are essential. A multidisciplinary approach is strongly recommended to enhance patient outcomes.
